# SOMAS: a platform for data-driven material discovery in redox flow battery development

**DOI:** 10.1038/s41597-022-01814-4

**Published:** 2022-12-01

**Authors:** Peiyuan Gao, Amity Andersen, Jonathan Sepulveda, Gihan U. Panapitiya, Aaron Hollas, Emily G. Saldanha, Vijayakumar Murugesan, Wei Wang

**Affiliations:** 1 Physical and Computational Sciences Directorate, Pacific Northwest National Laboratory, Richland, WA 99354 USA; 2 Environmental Molecular Sciences Laboratory, Pacific Northwest National Laboratory, Richland, WA 99354 USA; 3 Energy and Environment Directorate, Pacific Northwest National Laboratory, Richland, WA 99354 USA; 4 National Security Directorate, Pacific Northwest National Laboratory, Richland, WA 99354 USA

**Keywords:** Batteries, Batteries

## Abstract

Aqueous organic redox flow batteries offer an environmentally benign, tunable, and safe route to large-scale energy storage. The energy density is one of the key performance parameters of organic redox flow batteries, which critically depends on the solubility of the redox-active molecule in water. Prediction of aqueous solubility remains a challenge in chemistry. Recently, machine learning models have been developed for molecular properties prediction in chemistry and material science. The fidelity of a machine learning model critically depends on the diversity, accuracy, and abundancy of the training datasets. We build a comprehensive open access organic molecular database “Solubility of Organic Molecules in Aqueous Solution” (SOMAS) containing about 12,000 molecules that covers wider chemical and solubility regimes suitable for aqueous organic redox flow battery development efforts. In addition to experimental solubility, we also provide eight distinctive quantum descriptors including optimized geometry derived from high-throughput density functional theory calculations along with six molecular descriptors for each molecule. SOMAS builds a critical foundation for future efforts in artificial intelligence-based solubility prediction models.

## Abstract (summary)

**Table:** 

Measurement(s)	quantum descriptors • molecular descriptors • physical descriptors
Technology Type(s)	Computation • experiment

## Background & Summary

The aqueous solubility of organic molecules is a crucial property in multiple areas like synthesis chemistry, catalysis science, drug design, and energy science^1–4^. In energy science, to facilitate the rapid deployment of renewable energy, aqueous organic redox flow batteries (RFBs) have been increasingly recognized as a promising candidate for large-scale energy storage due to their inherent safety, potentially low-cost, and structure tunability^5,6^. In organic RFBs, the physicochemical properties of organic molecules significantly impact their performance characteristics^7^. The solubility of redox active organic species is a critical parameter in aqueous electrolyte design, as it determines the energy density of RFBs.

Versatility of organic molecular editing, in terms of both structural variations and functional group attachments, offers a unique possibility for artificial intelligence-based designing of highly soluble redox molecules for RFB application. However, the predictive understanding of the relationship between a functional property such as solubility and the chemical structure of organic molecules is lacking. Some structural and physiochemical parameters such as solvent accessible surface area (SASA) and acid dissociation constant (pKa) are known to influence the solvation process. Multiple physics-based models were developed using these properties, but the accuracy remains unsatisfactory^8–10^. Linear regression-based models, such as quantitative structure−property relationships (QSPRs) using molecular parameters also fail to produce reliable solubility predictions^11–13^. For example, the state-of-the-art models render prediction of solubilities with root-mean-square errors (RMSEs) of approximately 0.3−0.4 (log units) for simple organic molecules and 0.7−1.0 (log units) for drug molecules in small test sets^14^.

With recent development in both computer hardware and software, machine learning (ML) is increasingly being recognized as a powerful technique for material design and property prediction^15,16^. To develop generalizable and accurate ML models, large datasets with structural and chemical diversity of molecules with relevant quantum and molecular descriptors are extremely important. However, previous open-source solubility databases primarily designed for drug design are based on a few hundred drug molecules, which is very small and does not represent the relevant chemical parameter space of redox flow battery electrolytes. For example, the desired solubility of organic molecules in RFBs is much larger (≥0.5 M) than that of the drug molecules (<0.1 M). Also, strong acidic or basic organic molecules can be effective electrolyte in RFB, but most drug candidates are relatively weak acids and bases^17–19^. Therefore, organic RFB development efforts require a comprehensive database that covers relevant chemical parameter space.

**Fig. 1:**
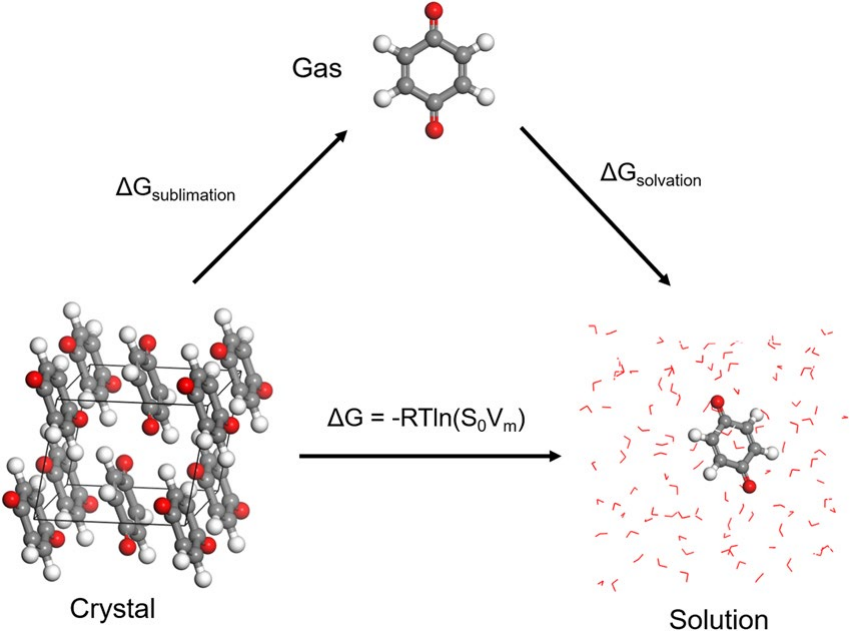
Thermodynamic cycle scheme of intrinsic solubility. $\Delta G=\Delta {G}_{sublimation}+\Delta {G}_{solvation}$. R, ideal gas constant, T, absolute temperature, S_0_, intrinsic solubility, V_m_, crystalline molar volume.

In this work, we build a comprehensive open access database “Solubility of Organic Molecules in Aqueous Solution” (SOMAS) that can serve as an optimal platform for developing aqueous solubility prediction models using ML methods. Unlike previous solubility databases, the SOMAS database focused only on neutral organic molecules and excluded organic salts and organometallic compounds to reduce data set bias in predictive models. Our database has a total of 11,696 organic compounds, which is nearly twice the number of organic compounds in AqSolDB, an open source database reported recently^20^. Of equal importance is that the number of molecules in the range of high solubility (>0.5 M) is also about two times more than AqSolDB database, providing a more comprehensive training dataset^20^. In addition to the experimental solubility, eight quantum descriptors derived from high-throughput density functional theory (DFT) calculations along with traditional molecular descriptors were also added to each molecule in the database, rendering it as an optimal platform for solubility prediction models relevant for RFB application. The choice of quantum and molecular descriptors are carefully selected to represent the thermodynamic cycle of aqueous solubility shown in Fig. 1. We curated the molecular data of experimental aqueous solubility with specific temperature, and literature references collected from a wide range of material/chemical engineering databases and published papers/handbooks. To reduce the rate of duplicate entries in the database, we implemented a new cross-validation method using independent molecular identifiers. Furthermore, the isomer structure is represented by the canonical isomeric Simplified Molecular Input Line Entry System (SMILES) string, which enables efficient tabulation and identification of stereoisomeric molecules with multiple chiral centers. Our data curation steps were designed to significantly improve the compatibility and accuracy of the machine learning model for complex structures such as chiral organic molecules. We expect SOMAS to serve the energy storage researchers and broader scientific community as an open source aqueous solubility dataset for training and benchmarking of ML and physics-based solubility models and pave the way for other physiochemical property predictions.

## Methods

**Fig. 2:**
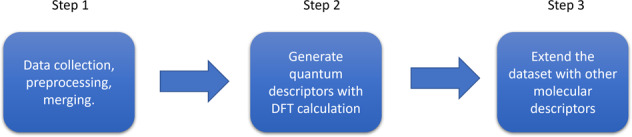
Workflow for database curation and augmentation of specific quantum and molecular descriptors.

We followed three steps to curate the database as illustrated in Fig. 2. First, we collected the molecular data of solubility from available aqueous solubility datasets and converted them to a standardized format. These separate data files were then combined into one single dataset. To reduce the proportion of duplicates in the database, the molecular data from various sources were cross validated by different identifiers. For a given molecule, if the solubility data from literatures are with large difference, we do not follow the previous average protocol^20^, as it is difficult to know the real weight function from different data sources. Instead, if the solubility difference of compounds with the same identifier is too large (>50%), the original references of the data are checked manually. Only the solubility value from the reliable source is reported if no clerical error is found in the original references (the grade of data source is shown in the following section). When data at multiple temperatures are available, only the solubility value which is the closest to room temperature is selected. After the curation of solubility data, high throughput DFT calculations were performed to generate the quantum descriptors for each molecule. Finally, several molecular descriptors were calculated using RDkit code (http://www.rdkit.org) and added to the merged dataset. The workflow is shown in Fig. 2.

### Step 1: Data pre-processing and merging

A set of three pre-processing steps were applied to each entry to format molecular representations and solubility values in the standardized units. The steps are as following:

#### Identifiers validation and unit conversion

**Fig. 3:**
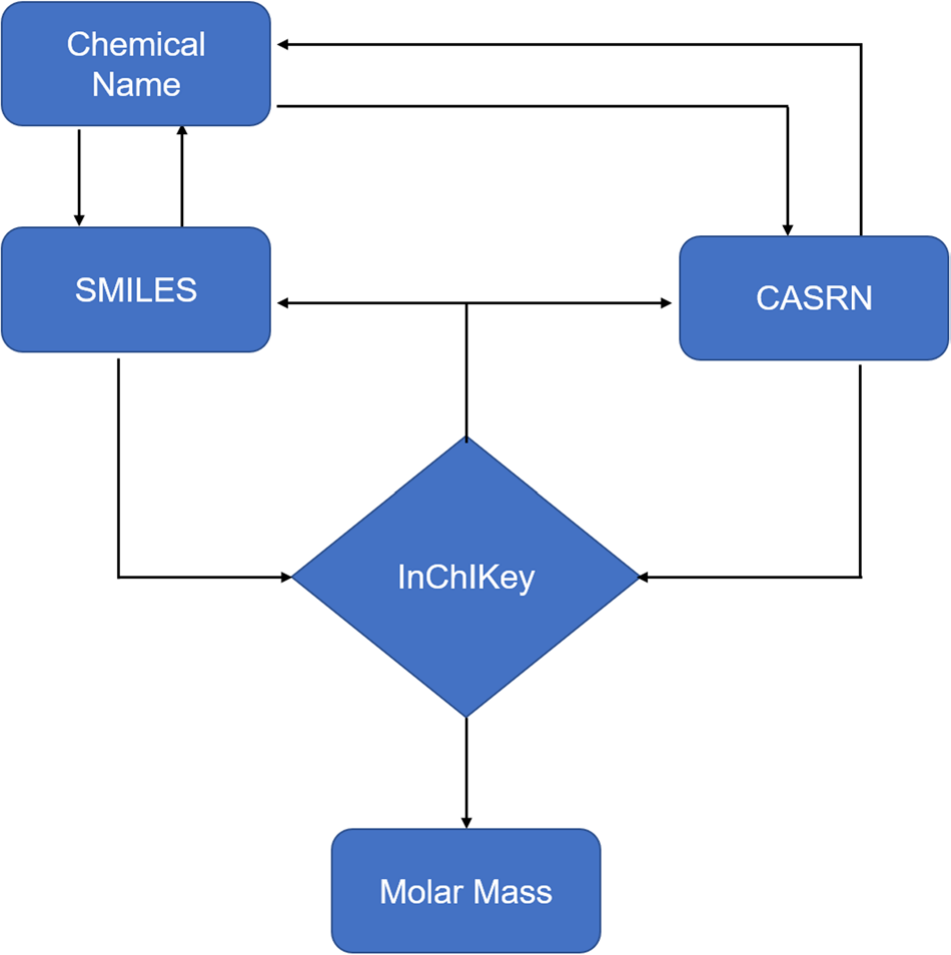
Workflow of identifiers cross-validation for molecules.

We selected four identifiers namely, chemical name, SMILES string, Chemical Abstracts Service Registry Number (CASRN), and International Chemical Identifier (InChIKey) to confirm the information of each molecular data with reduced duplication rate and enhanced cross-validation methods. Generally, two or more of the identifiers were identified for each molecule from different data sources. Molar mass, a common molecular descriptor, was also used as an additional identifier for curation. The workflow of molecule identification is shown in Fig. 3. InChIKey is used as the main identifier for compounds in our datasets, and no InChIKey collision of molecules has been observed in our databases. Although a single InChIKey could theoretically map to two or more InChI (International Chemical Identifier, an InChi string represents one molecule) strings, the possibility is rather small for a dataset with the size of less than 100,000 molecules^21^. In the absence of reported InChIKey, we used CASRN or the SMILES string to retrieve or construct an InChIKey. We used the following web sources to convert the CASRN to InChIKey: the Chemical Identifier Resolver web service of the National Cancer Institute (https://cactus.nci.nih.gov/chemical/structure), PubChem (https://pubchem.ncbi.nlm.nih.gov/), and ChemSpider (http://www.chemspider.com/). For the conversion of SMILES string to InChIKey, we have used the Open Babel^22^ and RDkit software. The unit of solubility in our database is mg/L. For solubility data with other units such as mol/L, it is calculated and converted to mg/L by the molar mass of molecule.

#### Isomeric SMILES string verification

To ensure validity and consistency of the SMILES strings, we have employed RDKit *mol* objects that converts SMILES to molecular structure. If an error occurred during the conversion, the SMILES strings were manually revised to correct the error. In addition, there is significant presence of chiral molecules in the database. In most of publications with previous SMILES string grammar, the chirality of molecule is not specified. In the Daylight SMILES notation, it allows the specification of configuration at tetrahedral centers, and double bond geometry with isomeric SMILES string which is also canonical. With the isomeric smiles, it can generate correct molecular configuration when converting the SMILES string to three-dimensional molecular model. It is reasonable to use common SMILES string for the molecule with one chiral center, as the solubility values or other physicochemical properties will be equal for the S and R isomers. However, if the molecule includes two or more chiral centers, the solubility values of all the isomers are often different from each other. Therefore, the isomer specification is very important to identify the accurate solubility data of molecules; however, it has been often ignored in previous databases. In our database, we compare the collected information as CASRN or InChIKey of the molecules with the existing result on PubChem and ChemSpider website. If its isomeric SMILES string is provided on these websites, the SMILES string from the original reference will be replaced by the isomeric SMILES string.

#### Racemic mixture removal

As mentioned in the last section, the isomers are not specified by isomeric SMILES strings in some previous papers and databases. Therefore, for a chiral molecule, the reported solubility value with common SMILES string could be one of its isomers or racemic mixture of isomers. The solubility values are usually not the same for all the isomers for molecules including two or more chiral centers. When the solubility data of a molecule exists for both a common SMILES string and a specified isomeric SMILE string, the solubility data of the molecule with the common SMILES string will be marked as racemic mixture and removed. Currently, 129 molecular data points of racemic mixture have been identified in our database. While we try to eliminate the racemic mixture in our database, in some cases, the original data source only provides the common SMILES as an identifier. Such molecular data are not able to be specified, although these data may be racemic mixture. These data are temporarily reserved in our database. And they will be replaced with the data of specified stereoisomer when the isomeric data are available. The stereoisomer number of these molecules is more than 2 and their InChIKey identifiers include the string “UHFFFAOYSA”.

#### Data sources

The solubility data were collected from six data sources. Table 1 presents the final size and identifier information of the six sources. The data from each data source is processed separately. The details of data source are listed below.

**Table 1:** Dataset sources list in the database.

Database	Final size	Compound Representation	Average molecular weight
1	1,068	Name, partial CASRN, SMILES	167.09
2	2,122	Name, partial CASRN,	216.23
3	149	Name, CASRN,	185.27
4	2,791	Partial Name, SMILES, partial CASRN	257.19
5	1,743	Name, CASRN	248.66
6	3,823	Name, InChIKey	266.28

Dataset 1: Dataset 1 is obtained by checking GDB-13 and GDB-17 datasets^23,24^ using EPA EPI suite (https://www.epa.gov/tsca-screening-tools/epi-suitetm-estimation-program-interface) and PubChem (https://pubchem.ncbi.nlm.nih.gov/).

Dataset 2: Dataset 2 is obtained by checking Chembl25 (https://www.ebi.ac.uk/chembl/) using EPA EPI suite. ChEMBL is a database of bioactive drug-like small molecules, it contains 2D structures, calculated properties (e.g. logP, Molecular Weight, Lipinski Parameters, etc.), and abstracted bioactivities (e.g. binding constants, pharmacology, and ADMET data). The data are abstracted and curated from the primary scientific literature.

Dataset 3: Dataset 3 is NIST Standard Reference Database 106 (NIST SRD106). NIST SRD106 is a database containing solubilities originally published in the IUPAC (International Union for Pure and Applied Chemistry) - NIST solubility data series.

Dataset 4: Dataset 4 is obtained from the Online Chemical modeling environment^25^ (OCHEM) (https://www.ochem.eu/home/show.do). The Online Chemical Modeling Environment is a web-based platform that aims to automate and simplify the typical steps required for QSAR modeling. It includes a large database of experimental measurements.

Dataset 5: Dataset 5 is obtained via eChemPortal (https://www.echemportal.org/echemportal/), which is an open source chemical property database developed by the Organization for Economic Co-operation and Development (OECD). Solubility data was extracted by searching experimental water solubility and temperature. We extracted the solubility data at 20–30 °C and its source link by changing the temperature filter from 20 to 30.

Dataset 6: Dataset 6 is obtained from Cui’s work published on *Frontiers in Oncology*^26^. The solubility data were obtained from ChemIDplus database (https://chem.nlm.nih.gov/chemidplus/) and Pubmed (https://pubmed.ncbi.nlm.nih.gov/) literature. The authors mentioned that these solubility data were measured at room temperature, but the temperature is not specified for every molecule in the original dataset. Note that we do not further check the reliability of temperature in this paper.

There are three grades in our database for the data sets. Data sets 3 and 5 are in grade 1, since the experimental method, detail of data fitting, and original reference are all provided. Data sets 1 and 2 are in grade 2 because only original reference is provided. Data sets 4 and 6 are in grade 3 as no specified original reference is provided.

### Step 2: Quantum descriptor generation

Eight solubility and redox potential related quantum descriptors, i.e., solvation energy, dipole moment, quadrupole moments, molecular volume, molecular surface area, the highest energy occupied molecular orbital (HOMO) energy, the lowest energy unoccupied molecular Orbital (LUMO) energy and molecular geometry are extracted from DFT calculations. DFT calculations were performed with the NWChem quantum chemistry package^27^. The initial 3D configurations of molecules are converted from SMILES string with the Experimental-Torsion “basic Knowledge” Distance Geometry (ETKDG) method^28^ and optimized by molecular mechanics method with the Universal Force Field (UFF) in the RDkit package. For some structures where RDKit could not render the 3D structure from the SMILES string, JMol’s SMILES to 3D structure interpreter was used (http://www.jmol.org/). These starting 3D molecular geometries were then optimized at the GFN2-xTB (semi-empirical extended tight binding) level of theory (or GFN-FF general force-field for relatively large 70 + atom molecules) with the CREST multi-level conformational search and optimizer software of Grimme and coworkers^29,30^. The analytical linearized Poisson-Boltzmann (ALPB) implicit solvation model for water was used in all CREST optimizations^31^. The optimized lowest energy conformers from CREST are subsequently optimized at the PBE0 level of theory^32,33^ with the 6–31 G** basis set^34–36^, except for the heavier elements Sb, Te, and I, which used the Stuttgart basis set and effective core potential (ECP)^37^. Long range dispersion interactions are corrected with the DFT-D3 method of Grimme and coworkers^38^. An effect of implicit water solvent with a dielectric constant of 78.4 is included via COnductor like Screening MOdel for Real Solvents (COSMO) model^39^. NWchem output file for each molecule will be made available upon request.

### Step 3: Molecular descriptor generation

Total of 1,826 Molecular descriptors, including 1,613 two-dimensional and 213 three-dimensional features can be generated using the Mordred package^40^ with RDkit. Only the SMILES string is required to calculate two-dimensional features whereas atomic coordinates along three cartesian axis are required to generate three-dimensional features. In this work, we only select several two-dimensional molecular descriptors that are related to solubility. They are calculated octanol-water partition coefficient, calculated molar refractivity, topological polar surface area, Labute’s Approximate Surface Area, Balaban’s *J* index, and Bertz CT index.

## Data Records

The SOMAS database consists of 11,696 organic compounds. The data are stored in the comma-separated values (CSV) file format and XYZ file. There are 26 columns in the *csv* file, i.e., identifiers, solubility, temperature, reference, data source reference, quantum descriptors (except optimized XYZ coordinates), molecular descriptors, and isomer information. The XYZ file is the optimized atomic coordinates by DFT calculation. All the XYZ files are compressed into a single *tar.gz* file. It includes five identifiers (CASRN, SMILES, Chemical name, InChIKey, Molar mass), eight quantum descriptors by DFT calculation (solvation energy, dipole moment, quadrupole moment, molecular volume, surface area, HOMO energy, LUMO energy, and optimized XYZ atomic coordinates) and six calculated molecular descriptors (Cal logP, Cal MR, TPSA, Labute ASA, Balaban J index, and Bertz CT index) data of all the molecules, as described in Table 2. The *csv* file (data.csv) and *tar.gz* file (XYZfiles.tar.gz) are accessible in Figshare repository (10.6084/m9.figshare.14552697)^41^.

**Table 2:** Topology of SOMAS database with selected descriptors.

Column Name	Description	Type
Name	Name of compound	String
SMILES	SMILES representation of compound	String
Molar mass	Molar mass (g/mol)	Float
CASRN	CAS registry number	String
Solubility	Experimental aqueous solubility value (mg/L)	Float
Temperature	Temperature (K)	Float
Reference	Source paper, book, or weblink for solubility value	String
Standard InChIKey	Hashed key of the IUPAC International Chemical Identifier	String
Data group	Data source	String
Isomer	Number of stereoisomers by RDkit	Integer
Solvation energy	COSMO based solvation energy by DFT calculation (kJ/mol)	Double
Dipole moment	Dipole moment by DFT calculation (Debye)	Double
Molecular volume	Volume of DFT optimized structure (Å^3^)	Float
Molecular surface area	Surface area of DFT optimized structure (Å^2^)	Float
Quadrupole Moment	Quadrupole Moment Asymmetry by DFT calculation (Buck)	Double array
E_HOMO	HOMO energy (eV)	Double
E_LUMO	LUMO energy (eV)	Double
E_gap	HOMO-LUMO energy gap (eV)	Double
Cal logP	Calculated octanol-water partition coefficient	Float
Cal MR	Calculated molar refractivity	Float
TPSA	Topological polar surface area	Float
Labute ASA	Labute’s approximate surface area	Float
Balaban J index	Balaban’s J index	Float
Bertz CT index	A topological complexity index of compound	Float

**Fig. 4:**
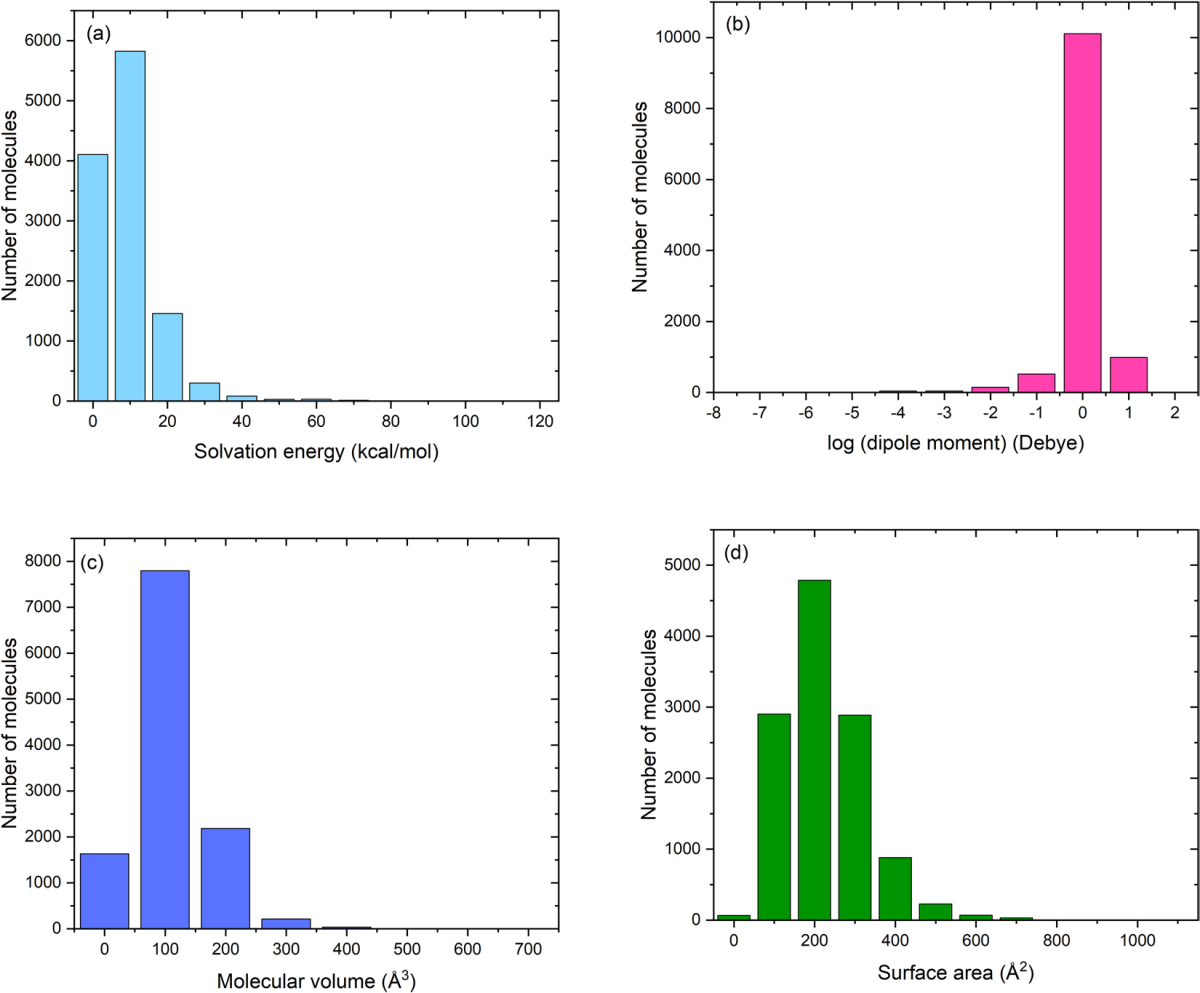
Distribution of solvation energy, dipole moment, molecular volume, and surface area obtained in DFT calculation. (**a**) Solvation energy. (**b**) dipole moment. (**c**) Molecular volume. (**d**) Surface area.

Figure 4 shows the distribution of solvation energy, dipole moment, molecular volume, and surface area of all molecules in SOMAS. It is observed that for most molecules, solvation energies are in the range of 0–20 kcal/mol, and dipole moment is primarily distributed at 1–10 Debye. The distributions of molecular volume and surface area lie within 100–200 Å^3^ and 200–300 Å^2^, respectively, indicating most of them are small molecules.

## Technical Validation

**Fig. 5:**
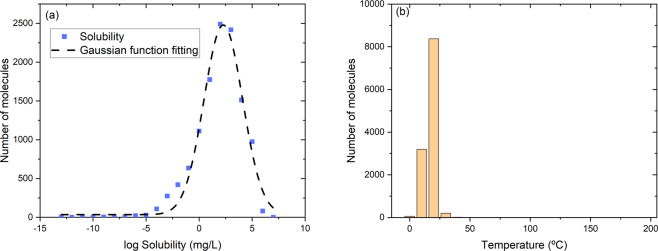
Distribution function of molecules as a function of log solubility (**a**) and distribution function of temperature (**b**) in SOMAS database. The dashed line represents the fitted Gaussian function.

The probability distribution of solubility is analyzed for technical validation. Figure 5(a) shows the number distribution of molecules as a function of log solubility. It is found that the distribution of solubility fits well with a Gaussian distribution, indicating that aqueous solubility is a multiscale phenomenon involving physical and chemical features of molecules. The peak center is located at 2–3 (100–1000 mg/L), which is consistent with typical range of solubility for organic molecules. As most of the original data sources do not provide experimental error information, it is difficult to estimate the error. A previous work provides a validation method that sorts by the occurrence frequency of the molecule^20^. However, the method only works for molecular data that are obtained from independent data sources. If the data in different databases are obtained from the same paper, the weight function will be overestimated. Therefore, in our database, we do not show the occurrence frequency of the molecule but instead provide original reference of paper or web link of each molecule to facilitate robust cross-validation. Although the solubility of an organic molecule is sensitive to temperature, most data are in the range of 10–30 °C (see Fig. 5(b)). Hence the temperature-related uncertainty is likely to be minimal for ML models. We would like to note that the database contains a few solubility data points that are extremely small (≤10^−10^ mg/L) or unrealistically high (>10^6^ mg/L) and should be treated with caution to avoid any data bias in predictive models. For example, extreme solubility data such as 1,000,000 mg/L is reported for some molecules that are likely to be miscible in water and often considered as infinite solubility.

**Fig. 6:**
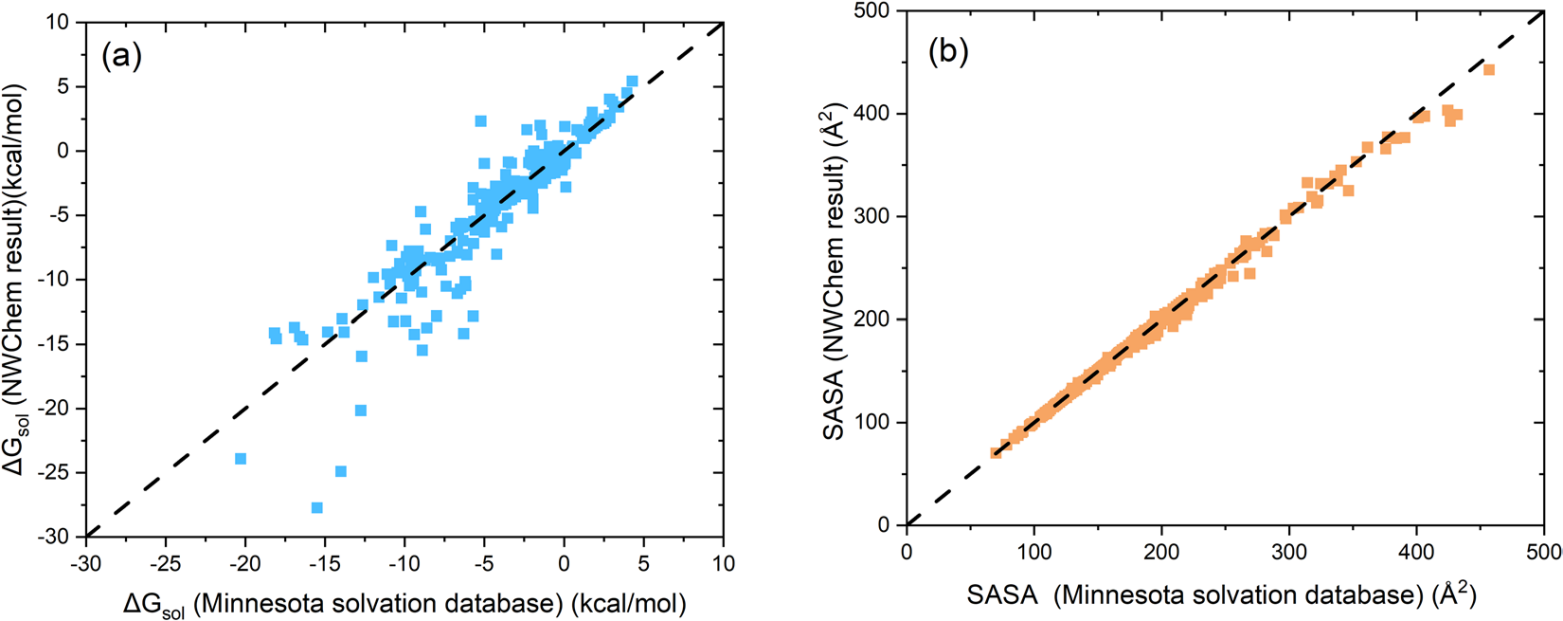
Comparison between experimental and calculated solvation free energy and SASA by COSMO-SMD model in NWChem. (**a**) solvation free energy. (**b**) SASA. The experimental solvation free energy data are extracted from Minnesota solvation database (https://comp.chem.umn.edu/mnsol/).

Table 2 shows quantum descriptors obtained from DFT calculations at the PBE0/6–31 G** level theory, which are proven to be accurate and reliable for describing intramolecular degrees of freedom and intermolecular interactions^33,42^. The solvation energy is calculated using COSMO model which is an implicit solvation model implemented in a number of quantum chemistry or semi-empirical codes such as Gaussian^43^, NWChem^27^, TURBOMOLE^44^, and Q-Chem^45^. To further evaluate our DFT-derived descriptors, we calculated the solvation free energies of 308 organic molecules with COSMO-SMD^46^ (Solvation Model Based on Density) model and compared with experimental values from Minnesota solvation database (https://comp.chem.umn.edu/mnsol/). As shown in Fig. 6a, the root mean square error (RMSE) of our COSMO-SMD calculation is 1.82, which is close to previous calculation result (1.42) by SMD^46^ with NWChem on 274 molecules from a subset of Minnesota solvation database. Also, the calculated solvent accessible surface areas (SASA) of 308 molecules are also in good agreement with Minnesota solvation database as shown in Fig. 6(b).

## Usage Notes

We present a comprehensive database comprising experimental aqueous solubility and solubility-related quantum and molecular descriptors of 11,696 organic molecules. The availability of the calculated quantum descriptors and molecular descriptors makes it possible to directly use the data for developing machine learning models. The SMILES and InChIKey representations of compounds as well as the atomic coordinates files are also provided as input for many machine learning codes. We recommend users to consider the temperature effect when using the data as training input to machine learning models.

## Data Availability

NWChem is distributed as open-source under the terms of the Educational Community License version 2.0 (https://www.nwchem-sw.org). The RDKit software is freely available under the BSD license (http://www.rdkit.org). The Open Babel software is freely available under GNU GPL (http://openbabel.org/wiki/Main_Page).
